# Posterior Occipitocervical Fusion for Unstable Upper Cervical Trauma in Old and Elderly Population, Although Decreases Upper Cervical Rotation, Does Not Significantly Increase Neck Disability Index

**DOI:** 10.1155/2020/7906985

**Published:** 2020-07-21

**Authors:** Panagiotis Korovessis, Vasileios Syrimpeis, Evangelia Mpountogianni, Ioannis Papaioannou, Vasileios Tsekouras

**Affiliations:** Orthopaedics Department, General Hospital of Patras, Patras 26335, Greece

## Abstract

**Background:**

Despite the research progress in the thoraco-lumbo-pelvic balance, cervical spine balance has only recently gained increasing interest. To our knowledge, there is a lack of research regarding sagittal occipitocervical spine balance restoration following posterior occipitocervical fusion (POCF).

**Purpose:**

The primary outcome measure is the evaluation of sagittal cervical alignment roentgenographic parameters and the secondary is the functional outcome (NDI), following POCF for upper (C1 & C2) cervical trauma (UCT) in coexistence with upper cervical spine degeneration. *Patients and Methods*. Twenty old and elderly patients aged 62 ± 12 years with evident upper cervical degeneration, who received POCF for upper C1 & C2 unstable cervical spine injuries, were included. C2-C7 lordosis, C2-C7 SVA, spinocranial angle (SCA), T1-slope, neck tilt (NT), thorax inlet angle (TIA), cervical tilt (CT), cranial tilt (CrT), and C0-C1 angle were measured. The subfusion angle was used to study the behavior of the unfused cervical segments below fusion. The Neck Disability Index (NDI) was used for the functional outcome evaluation. 29 age-matched individuals were used as controls for radiographic analysis and self-reported functional status comparison.

**Results:**

The roentgenographic data were measured 3 and 39 ± 12 months postoperatively. Twelve patients showed no disability, and eight showed mild disability. Postoperatively, the patients stood with less C2-C7 lordosis, SCA, and CT (*P* < 0.02) but with higher NT (*P* < 0.02) in comparison to the controls. The patient's neck disability (NDI) was increasing as TIA increases (*P*=0.023). Subfusion angle seems to adapt to C2-C7 lordosis (*P* < 0.0033) and C0-C2 angle (*P* < 0.003) without any changes till the last evaluation.

**Conclusions:**

POCF sufficiently restored occipitocervical sagittal balance along with functional outcome similar to controls in adult and elderly individuals with evident upper cervical degeneration. We do not recommend POCF for young active individuals without occipitocervical pathology, but in contrary, we recommend the removal of the spinocranial connection hardware after cervical fusion is completed.

## 1. Introduction

Posterior occipitocervical fusion (POCF) has been an effective surgical procedure for the treatment of occipitocervical and upper cervical instability (UCI) for a variety of pathologies (trauma, degeneration, etc.) [1–13]. POCF that is a demanding surgery, acts mechanically as a tension band required to promote immediately stability and subsequently permanent cervical fusion [14–16]. However, POCF restricts about 50% of the axial rotation and flexion-extension of the head and this is a significant disadvantage of this technique in young individuals without cervical degenerative disease [14–16]. POCF in inadequate sagittal position of the cervical spine, occipitocervical junction, and head may be associated with functional complications (loss/reduction of horizontal gaze, dysphagia, respiratory disturbance, and disability) [12, 17–24]. In contrary, POCF in the elderly with often significant degeneration of the upper cervical spine and occipitocervical junction is often beneficial for reducing pain and technical issues such as loss of screw fixation due to osteoporosis, pseudarthrosis, and significant bleeding in C1-C2 fixation [12, 17–24].

There is evidence that several roentgenographic parameters such as C0-C2 angle, T1-slope, C2-C7 SVA, and spinocranial angle (SCA) should be reconstructed with surgery to achieve good functional outcome [18–21].

There is a lack of information regarding the restoration of the occipitocervical spine and the impact of postoperative sagittal occipitocervical alignment in neck disability following UCI and POCF in adult and elderly population.

The primary outcome measure is the roentgenographic sagittal occipitocervical alignment and the secondary outcome measure is the neck disability following POCF for acute upper cervical injury (UCI).

## 2. Materials and Methods

The authors' institution is the single Level 1 trauma center covering spine trauma in a region of over 1.2 million people. After institutional review board approval was obtained, the authors reviewed the institutional database and patients' files for POCF from 2012 to 2015. Twenty consecutive patients, 16 males and 4 females, suffering from acute UCI who had underwent early successful POCF by one experienced senior orthopedic spine surgeon in this period were recruited for evaluation. POCF surgery was defined as the operation with evident radiological completed fusion and without serious mechanical complications that required reoperation. The patients' age at the time of surgery was averaged, SD 61 ± 12 years, ranging from 43 to 78 years (Table 1).

The inclusion criteria were adult (>40 years) and elderly patients, acute trauma, and symptomatic degeneration of upper cervical or occipitocervical spine. The exclusion criteria were patients with C1-C2 fusion, congenital instability, or previous spinal surgeries. Preoperative imaging included (a) plain roentgenograms and (b) CT/MRI scans. An age-matched control group of 29 consecutive asymptomatic individuals was selected without history of spine injury or operation. The control group was subsequently selected to match in age to the patients' group. The average, SD age of the 29 controls was 63 ± 14 years with a range of 44–76 years. On admission, 2 patients (10%) had incomplete spinal cord injury (ASIA Grades C). The most common indication for POCF was C2 Levine IIa and/or traumatic spondylolisthesis that were diagnosed in 10/20 (50%) patients (Table 1). Only supine preoperative roentgenograms of the cervical spine were available, while standing AP and lateral roentgenograms were used for the final postoperative evaluation. Standing anteroposterior (AP) and lateral roentgenograms of the cervical spine were taken in the age-matched controls.

The cervical trauma AO-classification [25] was used, and the validated national version of the Neck Disability Index (NDI) questionnaire [26] was filled out for all the 20 survived patients at the final evaluation.

### 2.1. Roentgenographic Study

**Figure 1:**
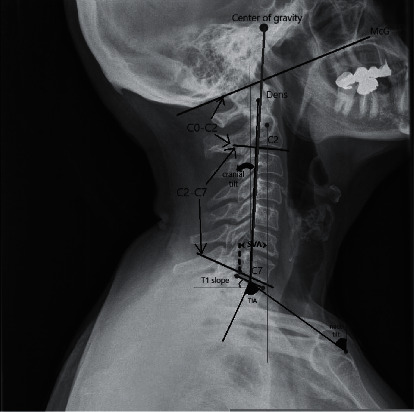
Lateral roentgenographic parameters used for sagittal cervical balance study.

Sagittal occipitocervical balance was evaluated in the patients with successful POCF and compared to that of the age-matched controls (Figure 1) [27–31] in terms of the following: (1) C0-C2 lordosis (the angle created by McGregor's line and the inferior surface of the axis); (2) C2-C7 lordosis (the angle between the lower plate of C2 and the lower plate of C7 vertebra); (3) spinocranial angle (SCA) (the angle between the C7-slope and the straight line joining the middle of the C7 end plate and the middle of the sella turcica); (4) T1-slope (the angle between an horizontal line and the superior endplate of T1); (5) C2-C7 SVA (the distance from the vertical line from the center of the C2 body and the posterior-superior corner of C7); (6) neck tilt (NT) (the angle formed by the reference vertical line drawn in the upper end of the sternum and a line connecting the center of the T1 upper endplate and the upper end of the sternum); (7) thorax inlet angle (TIA) (the angle formed by a line perpendicular to the superior endplate of T1 and a line connecting the T1 upper endplate and the upper end of the sternum); (8) cervical tilt (CT) (the angle between two lines, both originating from the center of the T1 upper endplate; one is vertical to the T1 upper endplate, and the other passes through the tip of the dens); (9) cranial tilt (CrT) (the angle between two lines, both originating from the center of the T1 upper endplate, with one passing through the dens and the other being a vertical line).

**Figure 2:**
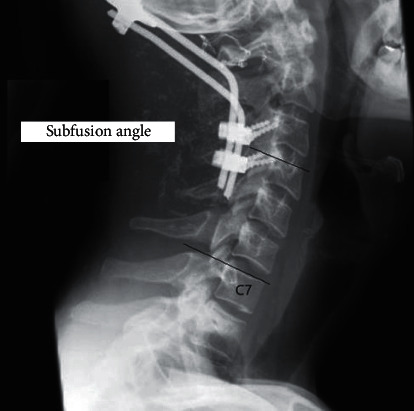
Lateral roentgenogram following POCF showing the subfusion angle measurement.

The subfusion angle (SA) (the angle formed by a line perpendicular to the superior endplate of the lowermost instrumented cervical vertebra and a line connecting the C7 upper endplate) was measured in the 3-month follow-up and at the final observation, in order to evaluate the adaptation of the nonfused cervical segments to POCF (Figure 2).

Fifteen randomly selected digital lateral radiographs from patients and controls were blindly measured twice within a one-week interval by two independent orthopedic surgeons. The reproducibility and repeatability of all roentgenographic measurements were evaluated using the kappa values. *P* values were tested against the significance level of 0.05.

### 2.2. Statistical Data Analysis

Data were analyzed using SPSS, statistics version 24 (Inc., Chicago, IL, USA). Continuous data were reported as mean ± SD.

The skewness and kurtosis tests were used to test the data frequency.

Levene's test of variance homogeneity was used to assess the equality of variances for each variable calculated for the two groups. The paired *t*-test was used for the comparison of the same continuous variable change. The bivariate Pearson correlation coefficient (*r*) was used to correlate different continuous roentgenographic and categorical variables. The categorical variables (gender, age, and NDI score) were graded in two groups: gender (women: 0, men: 1); age groups in controls (≤66 years: 0, >66 years: 1) and age groups in patients (≤61 years: 0, >61 years: 1). The ages of 66 and 61 years old were chosen because they were the average age of controls and patients, respectively. No disability (NDI score: 0–4) was graded (0) and mild disability (NDI > 4) was graded (1) at the final observation. The NDI scores in the patients at the final observation were tested for any significant correlation with each of the roentgenographic variables.

## 3. Results

There were no skewed data and no kurtosis issues while Levene's heterogeneity test showed homogeneity for all variables at baseline in both groups. The kappa values for both inter- and intraobserver agreements for the roentgenographic parameters measuring were 0.98–1.

The time elapsed between the trauma and the day of surgery ranged from 1 to 3 days (average: 1.4 days). The surgery duration averaged to 105 min (range: 90–120 min). The average hospital stay was 7 days (range: 3–21 days), although this was decreased to 3 days (range: 2–4 days) in cases of isolated UCI.

The follow-up observation averaged to 39 ± 12 (range: 25–57 months).

### 3.1. Functional Results

The average NDI score was 10% ± 8% (range: 2–24%). Twelve patients showed no disability (NDI: 0–8%), while 8 patients reported mild disability (10–28%).

### 3.2. Roentgenographic Results

**Figure 3:**
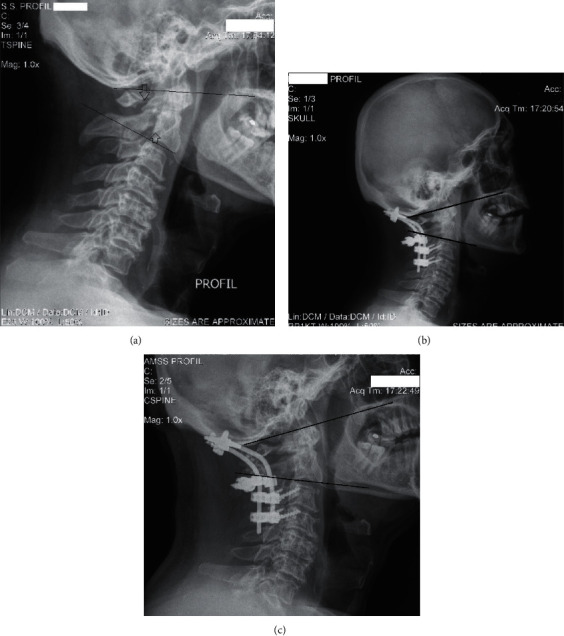
(a) Lateral roentgenogram of a 61-year-old man with C2 Levine IIb traumatic spondylolisthesis with >5 mm diastasis (lower arrow) and comminuted Jefferson fracture (upper arrow). The degeneration in the upper most cervical spine is evident. (b) Postoperative lateral roentgenogram following surgery showing reduction of the injury and POCF. There is an improved lordotic C0-C2 angle compared to the preoperative angle in (a). (c) Follow-up observation 36 months postoperatively showing a complete spinal fusion and a lordotic C0-C2 angle.

There were no radiological changes in any sagittal roentgenographic parameter value between 3 months postoperatively and at the last observation (Figures 3(a)–3(c)).

Controls stood with increased C2-C7 lordosis, SCA, and CT (*P*=0.019, <0.003, and <0.001, respectively) compared to their counterparts (patients), whereas patients showed higher NDI than controls postoperatively (*P*=0.013).

Significant correlations were shown between anthropometric and roentgenographic parameters in both groups (Tables 2 and 3).

Female controls stood with more C2-C7 lordosis and SCA than their male counterparts (*P*=0.008 and 0.017, respectively), while C2-C7 SVA was greater in men (*P*=0.014). TIA was greater in controls aged ≥66 years (*P*=0.041) than those aged <66 years.

Patients with no disability showed less TIA than those with mild disability (ANOVA, *P*=0.023).

The subfusion angle (SA) that averaged 0.65 ± 13° three months postoperatively did not change at the last observation (2.6 ± 14°) (paired *t*-test, *P*=0.76).

SCA is increasing with increasing C2-C7 lordosis (*P* = 0.0022 and 0.0001) and C0-C2 angle (*P* = 0.019 and 0.024). In contrary, SCA is decreasing with increasing T1-slope (*P* = 0.038) and CT (*P* = 0.016 and 0.018) (Table 4).

## 4. Discussion

Instability in the occipitocervical junction from different causes (degenerative disease, trauma, etc.) had been, for a long period, the primary indication for POCF [32–35] and has been successfully used in adults and the elderly suffering from unstable UCI, degenerative upper cervical spine, and inflammatory C1-C2 instability [10, 11, 13, 36, 37].

In this cohort, POCF restored immediately and maintained sufficiently the sagittal cervical and occipitocervical alignment. This correction was associated with low or no neck disability.

Contemporary POCF includes plate-screw-rod constructs that provide immediate postoperative stability [37, 38]. However, POCF in young adults reduces the motion of the C0 upper cervical spine, and subsequently, “occipitium sparing” posterior fusion techniques have been introduced by some authors [14, 33–36]. On the other hand, other authors postulated that the “occipitium sparing” surgery with C1-C2 fixation exposes a patient at a potential of highly increased intraoperative bleeding, while the sacrifice of the degenerated C0 upper cervical spine junction in adult or even elderly people may not make a significant clinical difference in the final functional outcome [33–36]. The aforementioned was also shown in these series where 12 and 8 adult and elderly patients had no or mild disability despite the occipitocervical immobilization.

There is a paucity of data regarding the sagittal occipitocervical parameters that determine good and pertinent clinical outcomes in old and elderly patients undergoing POCF for fresh unstable UCI.

The cervical spine is remarkably mobile and adapts its sagittal alignment to that of the thoracolumbar spine in order to maintain horizontal gaze [38–44]. Because of these adaptation movements, the physiological cervical spine in asymptomatic populations may not be necessarily lordotic but rather flat (45.8%) or kyphotic (21.7–33%) [19, 20, 41–44]. In this study, C2-C7lordosis averaged to −22 ± 15° in the controls, and that is significantly more lordotic than the −9.9 ± 20° that was measured in our patients (*P* = 0.019).

C0-C2 angle expresses the functional and balanced position of the head on the cervical spine in which an individual feels most comfortable [45]. In this study, patients showed postoperatively a lordotic C0-C2 angle close to that of the age-matched controls, indicating a sufficient restoration of the sagittal balance in upper cervical spine. The lordotic C0-C2 angle was achieved by the position of the patient's head during surgery, the appropriate contouring of the rods connecting the scull with the upper cervical spine and the intraoperative control of the desired occipitocervical junction position using an image intensifier [30, 31]. It is recommended that the C0-C2 angle to be set even more lordotic than the preoperative value in order to avoid dysphagia and respiratory disturbance [17, 22]. Because of the trauma mechanism, no preoperative standing lateral roentgenograms of the patients were available in order to make such comparisons.

In this study patients, C2-C7 lordosis was increased as the C0-C2 angle was increasing, indicating that the fixed C0-C2 angle following POCF “forces” the free segments below fusion to adapt their sagittal alignment to restore via the POCF C0-C2 angle.

In the recent literature, the most important parameter for good clinical outcomes following cervical surgery is to get T1-slope <40° [18–20]. In this study's patients, the T1-slope was <40° and it did not differ (*P* = 0.31) from the controls' T1-slope. Previous studies [18, 19, 44] showed that T1-slope increases as thoracic kyphosis increases, compensatory to maintain horizontal gaze, and this change affects the C2-C7lordosis. In our patients, T1-slope was positively correlated with C2-C7 lordosis (*P* = 0.006), which possibly indicates an adaptation of C2-C7 lordosis to preexisted thoracic kyphosis.

All cervical spines in both patients and controls were well balanced in the sagittal plane since C2-C7 SVA was <40 mm [46].

Chen et al. [20] showed that both T1-slope (>40 mm) and C2-C7 SVA negatively influence functional outcome scores (ED, 5Q, and HRQOL) in asymptomatic population [46]. Iver et al [47] showed that high C2-C7 SVA and low T1-slope are independent predictors of the high preoperative NDI score. NDI scores were not measured in our asymptomatic controls, but in our patients there was observed a tendency for high NDI scores with increasing T1-slope (*P* = 0.097). In this series, the postoperative NDI did not correlate with C2-C7SVA (*P* = 0.5). We speculate that this finding may explain the low disability (NDI <12) scores in our patients.

The center of gravity of the head is located at the posterior corner of the sella turcica, and therefore, SCA is a good marker to analyze the head positioning [46, 48, 49]. Some authors [19, 46, 50] reported that SCA averages to 83° ± 9° in symptomatic population and is correlated with the cervical lordosis [50] while the combination of C7 or T1-slope < 40° with an SCA (83 ± 9°) was associated with an economical balance [19]. In our patients, the SCA was 75.9 ± 7°, close to the economical balance limits, previously reported [19].

Lee et al. [29] described the TIA as the sum of ΝΤ + Τ1-slope in patients with the ankylosing cervical spine, but later, Janusz et al [39] postulated that TIA is not accurate in patients with symptomatic cervical degenerative disease. This was also justified in our patients, where TIA was postoperatively only 50% of the sum of ΝΤ + Τ1-slope. Our study showed that the functional outcome measure (NDI) in our patients was better in individuals with low TIA angle (*P* = 0.023).

The subfusion angle (SA), which was defined and used first in this study, seems to adapt to the C2-C7lordosis and C0-C2 angle, and it remained unchanged till the final observation indicating no subadjacent segment degeneration.

Only few studies reported on the complications following POCF for pure unstable traumatic cervical injuries [17, 21, 22, 34]. The reported complication rate averages to 52% in patients who received POCF for several causes [23, 30, 51]. A serious complication following UCI and POCF is the airway compromise (4.9%) that is thought to occur secondary to retropharyngeal swelling [51, 52]. No patient in our series showed airway compromise. Another serious complication, the dysphagia [46, 52] following POCF, has a different mechanism than that in anterior cervical surgery. Some authors supported the hypothesis of mechanical stenosis of the airway because of loss of the lordotic C0-C2 angle and suggested the correction of this angle to the lordotic preoperative neutral position or to a slightly more lordotic angle, since dysphagia occurs following POCF [22, 46, 53, 54]. In our series, there was no patient with dysphagia, probably because the C0-C2 angle in the operated patients was sufficiently restored.

The reported screw failure following POCF in nontraumatic instability cases was 7–12.5% [12, 43]. In this series, we did not include patients with loss of occipital plate fixation and/or revision surgery.

There are few limitations in this study: (1) the retrospective design; (2) the relative small number of patients; (3) the lack of standing preoperative roentgenograms for comparison because of the trauma mechanism; (4) postoperative restriction of the head and uppermost cervical spine; and (5) nearly half of the patient who underwent POCF were younger than 60 years.

Because of the lack of preoperative standing X-rays, we used a control group for a more reliable comparison. Supine roentgenograms in the controls would have led us to scientifically not sound results and conclusions, since all spine balance comparisons are currently routinely made in standing position only.

The advantages of this study are as follows: (1) the one surgeon's consecutive series, (2) the homogenous sample of trauma cases, and (3) the age-matched controls for comparison.

We do not advocate POCF in young active individuals without occipitocervical acute or other pathology where a fusion sparing surgical technique is recommended. Since 2017, in our department, we routinely remove the craniocervical hardware (occipital plate, screws, and cranial end of the rods) after radiological cervical fusion is achieved, in young patients, without upper cervical spine symptomatic degenerative disease.

The most important sagittal cervical balance roentgenographic parameters, e.g., C0-C2 lordosis, T1-slope, C2-C7 SVA, TIA, and SCA that are responsible for the clinical outcome measuring, were postoperatively restored and remained till the last evaluation within the “normal” limits.

In conclusion, POCF resulted in sufficient restoration of the sagittal cervical balance, as compared to age-matched controls, without simultaneous decrease of the neck disability score.

## Figures and Tables

**Table 1:** Demographic data of 20 patients that received occipitocervical fusion for upper cervical spine injuries.

ID	Gender	Age	Surgery diagnosis	Levels of fusion	Surgical complications	Follow-up in months
1	M	58	C2 D'Alonzotype 2	C0-C4	Superficial infection	39
					Surgical debridement	
2	M	61	C2 Levine IIb	C0-C4	No	31
			C2 traumatic spondylolisthesis			
3	F	75	C2 D'Alonzotype 2	C0-C4	No	52
4	M	49	C2 D'Alonzotype 3	C0-C3	No	25
5	M	51	C2 Levine IIa	C0-C3	No	26
			C2 traumatic spondylolisthesis			
6	F	72	C2 D'Alonzotype 2	C0-C5	No	28
7	F	71	C1 IIIa/AO	C0-C3	No	24
8	M	75	C1 IIIa/AO	C0-C3	No	25
9	M	78	C2 Levine IIa	C0-C4	No	29
			C2 traumatic spondylolisthesis			
10	M	56	C2 Levine II	C0-C4	No	41
			C2 traumatic spondylolisthesis			
			C2 traumatic spondylolisthesis			
11	M	68	C2 Levine II	C0-C5	No	56
			C2 traumatic spondylolisthesis			
12	M	60	C1 IIIa/AO	C0-C3	Superficial infection	27
					Surgical debridement	
13	M	44	C1 IIIb/AO	C0-C7	No	39
14	M	60	C2 Levine IIa	C0-C3	No	47
			C2 traumatic spondylolisthesis			
15	M	38	C2 Levine IIa	C0-C5	Νo	54
			C2 traumatic spondylolisthesis			
16	F	58	C2 Levine IIa	C0-C4	No	52
			C2 traumatic spondylolisthesis			
17	M	60	C1 IIIa/AO	C0-C4	No	57
18	M	50	C2 Levine IIa	C0-C4	No	49
			C2 traumatic spondylolisthesis			
19	M	75	C2 D'Alonzotype 3	C0-C4	Νo	53
20	M	69	C2 D'Alonzotype 3	C0-C6	Superficial infection	25

**Table 2:** Correlation matrix between age, gender, and roentgenographic parameters in 29 asymptomatic individuals.

Parameters	Gender	C2-C7 lordosis	T1-slope	Spinocranial angle	C2-C7 SVA	Neck tilt	Thorax inlet angle	Cervical tilt	Cranial tilt	C0-C2 angle
Age	*r* = −0.169, *P*=0.382	**r** **=** **−0.470**, **P**=0.01	**r** **=** **0.452**, **P**=0.014	*r* = −0.174, *P*=0.365	*r* = 0.244, *P* = 0.201	*r* = 0.077, *P* = 0.693	**r** **=** **0.450**, **P** = **0.014**	*r* = 0.357, *P* = 0.057	*r* = 0.293, *P* = 0.122	*r* = 0.021, *P* = 0.914
Gender	1	**r** **=** **0.435**, **P**=0.018	*r* = 0.094, *P*=0.628	**r** **=** **−0.441**, **P**=0.017	***r*** **=** **0.453,P** **=** **0.014**	***r*** **=** **0.109**, **P** = **0.575**	*r* = 0.158, *P* = 0.412	**r** **=** **−163**, **P** **=** **0.399**	**r** **=** **0.354**, **P** **=** **0.059**	**r** **=** **0.258**, **P** **=** **0.177**
C2-C7 lordosis		1	*r* = −0.481, *P*=0.08	*r* = −0.12, *P* = 0.269	*r* = 0.161, *P* = 0.403	*r* = 0.215, *P* = 0.264	*r* = −0.271, *P* = 0.155	**r** **=** **−667**, **P** ≤ **0.001**	*r* = 0.043, *P* = 0.823	**r** **=** **0.482**, **P** **=** **0.008**
T1-slope			1	*r* = −0.348, *P* = 0.06	**r** **=** **0.468**, **P** **=** **0.011**	*r* = −0.210, *P* = 0.273	**r** **=** **0.728**, **P** **≤** **0.001**	**r** **=** **0.790**, **P** **≤** **0.001**	**r** **=** **0.649**, **P** **≤** **0.001**	*r* = −0.059, *P* = 0.762
Spinocranial angle				1	**r** **=** **−0.663**, **P** **≤** **0.001**	*r* = −0.172, *P* = 0.372	**r** **=** **−0.426**, **P** **=** **0.021**	*r* = 0.102, *P* = 0.6	**r** **=** **−0.693**, *P* **≤** **0.001**	**r** **=** **−0.407**, **P** **=** **0.029**
C2-C7 SVA					1	*r* = 0.230, *P* = 0.229	**r** **=** **0.571**, **P** **=** **0.001**	*r* = −0.114, *P* = 0.557	**r** **=** **0.902**, *P* **≤** **0.001**	*r* = 0.204, *P* = 0.288
Neck tilt						1	**r** **=** **0.517**, **P** **=** **0.04**	*r* = −0.361, *P* = 0.055	*r* = 0.104, *P* = 0.591	*r* = 0.173, *P* = 0.368
Thorax inlet angle							1	**r** **=** **0.439**, **P** **=** **0.017**	**r** **=** **0.642**, *P* **≤** **0.001**	*r* = 0.173, *P* = 0.368
Cervical tilt								1	*r* = 0.046, *P* = 0.82	*r* = −0.294, *P* = 0.121
Cranial tilt									1	*r* = 0.269, *P* = 0.269

The results in bold present statistical significance with *P* < 0.05; *r* = Pearson correlation coefficient; *P* = probability value.

**Table 3:** Correlation matrix between age, gender, and final follow-up roentgenographic parameters in 20 operated patients.

Parameters	Age	C2-C7 lordosis	Spinocranial angle	T1-slope	C2-C7 SVA	Neck tilt	Thorax inlet angle	Cervical tilt	Cranial tilt	C0-C2 angle
Gender	*r* = 0.340, *P*= 0.182	*r* = 0.131, *P* = 0.616	*r* = 0.179, *P* = 0.492	*r* = −0.026, *P* = 0.921	*r* = −0.148, *P* = 0.572	*r* = −0.178, *P* = 0.493	*r* = -0.163, *P* = 0.533	**r** **=** **0.124**, **P** **=** **−0.636**	*r* = −0.230, *P* = 0.374	*r* = −0.111, *P* = 0.672
Age	1	*r* = −0.107, *P* = 0.682	*r* = −0.022, *P* = 0.933	*r* = 0.284, *P* = 0.270	*r* = 0.070, *P* = 0.791	*r* = 0.020, *P* = 0.940	*r* = 0.304, *P* = 0.236	*r* = 0.291, *P* = 0.256	*r* = −0.014, *P* = 0.957	*r* = 0.263, *P* = 0.307
C2-C7 lordosis		1	*r* = −0.77, *P* = 0.770	**r** **=** **−0.638**, **P** **=** **0.006**	*r* = 0.468, *P* = 0.058	*r* = 0.213, *P* = 0.412	**r** **=** **−0.487**, **P** **=** **0.047**	**r** **=** **−0.663**, **P** **=** **0.004**	*r* = 0.044, *P* = 0.866	**r** **=** **0.555**, **P** **=** **0.021**
Spinocranial angle			1	*r* = −0.412, *P* = 0.101	*r* = −0.068, *P* = 0.796	*r* = 0.383, *P* = 0.130	*r* = −0.127, *P* = 0.626	*r* = 0.051, **P** = 0.847	**r** **=** **−0.708**, **P** **=** **0.001**	*r* = −0.231, *P* = 0.372
T1-slope				1	*r* = 0.016, *P* = 0.950	*r* = −0.398, *P* = 0.114	**r** **=** **0.714**, **P** **=** **0.001**	**r** **=** **0.788**, **P** **≤** **0.001**	*r* = 0.319, *P* = 0.213	*r* = −0.265, *P* = 0.304
C2-C7 SVA					1	*r* = −0.123, *P* = 0.637	*r* = −0.077, *P* = 0.768	*r* = −0.255, *P* = 0.238	*r* = 0.414, *P* = 0.099	*r* = 0.123, *P* = 0.637
Neck tilt						1	*r* = 0.357, *P* = 0.159	*r* = −0.106, *P* = 0.686	*r* = −0.447, *P* = 0.072	*r* = 0.247, *P*= 0.339
Thorax inlet angle							1	**r** **=** **0.766**, **P** **≤** **0.001**	*r* = −0.16, *P* = 0.951	*r* = −0.081, *P* = 0.757
Cervical tilt								1	*r* = −0.333, *P* = 0.192	*r* = −0.336, *P* = 0.187
Cranial tilt									1	*r* = 0.112, *P* = 0.668

The results in bold present statistical significance with *P* < 0.05; *r* = Pearson correlation coefficient; *P* = probability value.

**Table 4:** Pearson correlation matrix between roentgenographic parameters and the subfusion angle 3 months postoperatively and in the last follow-up, in 20 operated patients.

Parameters	Subfusion lordosis 3 months postoperatively	Subfusion lordosis in the last follow-up
C2-C7 lordosis	**r** **=** **0.705**, *P* **=** **0.0022**	**r** **=** **0.816**, *P* **=** **0.0001**
SCA	*r* = −0.333, *P* = 0.208	*r* = −0.083, *P* = 0.761
T1-slope	*r* = −0.325, *P* = 0.219	**r** **=** **−0.521**, *P* **=** **0.038**
C2-C7 SVA	*r* = 0.369, *P* = 0.160	*r* = 0.174, *P* = 0.519
NT	*r* = −0.341, *P* = 0.197	*r* = −0.070, *P* = 0.797
TIA	*r* = −0.495, *P* = 0.122	*r* = −0.364, *P* = 0.271
CT	**r** **=** **−0.698**, *P* **=** **0.016**	**r** **=** **−0.693**, *P* **=** **0.018**
CrT	*r* = 0.223, *P* = 0.406	*r* = 0.050, *P* = 0.854
C0-C2 angle	**r** **=** **0.78**, *P* **=** **0.019**	**r** **=** **0.559**, *P* **=** **0.024**

The results in bold present statistical significance with *P* < 0.05; SCA: spinocranial angle; NT: neck tilt; TIA: thorax inlet angle; CT: cervical tilt; CrT: cranial tilt; *r* = Pearson correlation coefficient; *P* = probability value.

## Data Availability

All the data used in the study are available from the corresponding author through the Hospital Electronic Patient Record System
